# From Biological Mechanisms to Clinical Outcomes: A Scoping Review Comparing Immediate and Delayed Dental Implant Placement Protocols

**DOI:** 10.3390/jcm15020682

**Published:** 2026-01-14

**Authors:** Nuttaya Phrai-in, Pimduen Rungsiyakull, Aetas Amponnawarat, Apichai Yavirach

**Affiliations:** 1 Department of Prosthodontics, Faculty of Dentistry, Chiang Mai University, Chiang Mai 50200, Thailand; nuttaya_phr@cmu.ac.th (N.P.-i.); pimduen.rungsiyakull@cmu.ac.th (P.R.); 2 Department of Advanced General Dentistry and Dental Public Health, Faculty of Dentistry, Chiang Mai University, Chiang Mai 50200, Thailand; aetas.a@cmu.ac.th

**Keywords:** osseointegration, biomarkers, wound healing, foreign body reaction, titanium implant, placement protocol

## Abstract

**Background/Objectives:** Dental implant placement protocols including immediate (IIP) and delayed implant placement (DIP) are likely to affect bone tissue repair and regeneration after the surgery. Despite many benefits of IIP, it has remained unclear whether IIP demonstrates comparable healing processes and outcomes to those observed in DIP. This review aims to summarize and compare biological and clinical outcomes of IIP and DIP, focusing on success and survival rates, periodontal status, esthetics and radiographic outcomes, and biochemical markers. **Methods:** A literature search of electronic databases was conducted using PubMed/MEDLINE, Embase, and the Scopus databases (January 1983–February 2025). 109 articles published in English, consisting of in vitro, in vivo, and clinical studies met the inclusion criteria. **Results:** This review shows that both IIP and DIP show similar implant survival rates, but IIP may lead to a higher risk of mid-facial recession in esthetic areas. DIP, on the other hand, can result in better soft tissue and bone healing. Histological and radiographic evidence shows comparable bone to implant contact (BIC) between the two methods, although peri-implant bone loss tends to be higher with IIP. Lastly, although specific molecular markers are well-established in all phases of osseointegration following DIP, there is no available literature comparing differences in biomarkers during healing periods between IIP and DIP. **Conclusions:** This review highlights the similarities and differences in the outcomes of IIP and DIP, as well as the knowledge gaps that require further investigation, providing valuable insights for predicting treatment outcomes and managing complications associated with dental implant placement.

## 1. Introduction

Preserving optimal oral health, especially preserving natural teeth, is important. Despite this, tooth loss remains common, particularly among the elderly. In 2019, the prevalence of edentulism in adults over 60 in the Western Pacific Region ranged from 12% to 37.7% [1], and in 2022, the WHO estimated a global prevalence of 23% in this age group [2]. Replacing missing teeth is a clinical challenge, requiring comprehensive approaches that address both functional and esthetic. Over recent decades, dental implants have become a well-documented and reliable treatment option, offering superior functional and esthetic outcomes compared with conventional dentures [3,4,5,6,7]. Implant success depends on various factors, including material biocompatibility, implant surface topography, design, bone quality, patient health, surgical techniques, and loading conditions [8,9]. While these factors are well understood, the influence of different implant placement protocols on peri-implant bone healing and clinical outcomes remains unclear.

Whether different dental implant placement protocols affect bone tissue repair and regeneration has become an increasingly intriguing topic in the field. The original implant placement protocol, termed delayed implant placement (DIP), requires placing an implant into a completely healed extraction socket, followed by another 3–6 months of osseointegration period before occlusal force loading [10]. The entire process takes approximately one year, leaving the patients edentulous for an extended period of time. This necessitates meticulous consideration of the implications associated with prolonged tooth loss and may give rise to esthetic concerns during treatment procedures. Hence, a new method has been proposed to facilitate the placement of dental implants immediately after tooth extraction to reduce the number of surgical operations and shorten the overall duration of treatment. This technique is termed immediate implant placement (IIP).

In recent decades, IIP has become more popular despite being supported by relatively fewer studies compared to DIP, as it was introduced later. Several studies have compared the success and survival rates of implants between IIP and DIP; however, the reliability of these results is limited due to heterogeneity and inconsistent definitions of success rates across studies. Furthermore, various outcomes regarding bone tissue repair and regeneration such as periodontal status, esthetic outcomes, and biological responses, have remained insufficient. To explore the dynamics of healing after immediate implant placement (IIP), assessing changes in biomarker levels at each stage of healing may provide valuable insights. Peri-implant crevicular fluid (PICF) is a site-specific biofluid that can be analyzed noninvasively to monitor these biomarkers. Tracking PICF biomarker changes has emerged as a promising method for the early detection of peri-implant tissue pathology. However, the findings on alterations in biomarkers and molecular dynamics associated with IIP and DIP, which may potentially be beneficial for predicting treatment outcomes and managing complications, have not been thoroughly investigated. Consequently, it has remained unclear whether IIP demonstrates comparable healing processes and outcomes to those observed in DIP. While most existing reviews focus on isolated clinical outcomes and provide limited integration with the underlying biological mechanisms, the present review adopts an integrative approach by synthesizing evidence from preclinical and clinical studies to link biological processes with clinical outcomes. This framework aims to enhance the understanding of IIP from a biological perspective and to inform strategies for overcoming current clinical challenges. Therefore, the objectives of this scoping review are (1) to review and summarize the study methods and strategies used to measure parameters related to the success and survival rates of implants in in vitro, in vivo, and clinical settings and (2) to compare the outcomes across various aspects of IIP and DIP based on the findings from in vitro, in vivo, and clinical studies to date.

## 2. Materials and Methods

**Figure 1:**
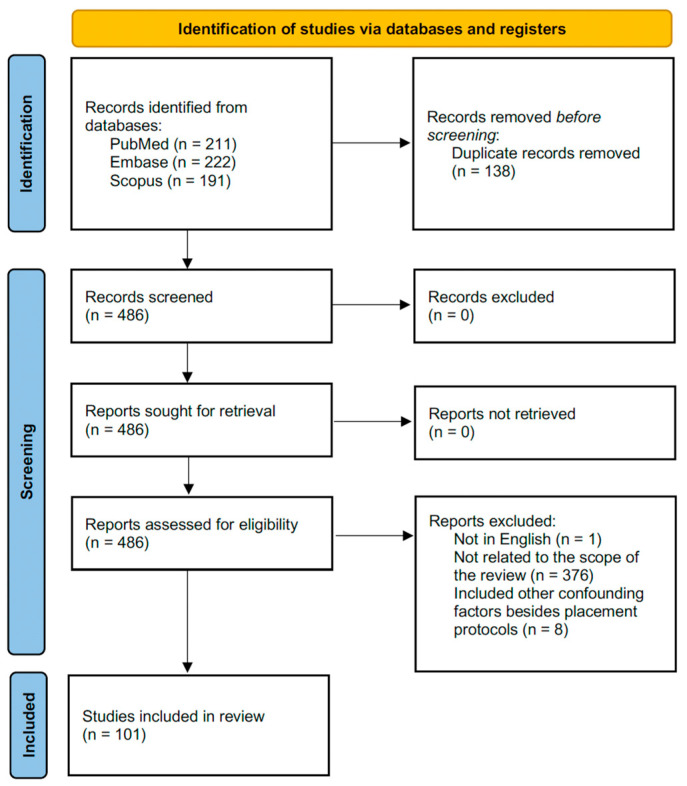
PRISMA flow diagram of this scoping review.

This scoping review aims to include studies on timing of dental implant placement outlined in the contemporary literature. The objectives are to identify the measurement strategies and differences in various outcomes between IIP and DIP, as well as to provide recommendations for clinical decision-making that support evidence-based practice. As shown in Figure 1, a literature search of electronic databases was conducted using PubMed/MEDLINE, Embase, and the Scopus databases from January 1983 to February 2025. The search keywords including combinations of terms related to dental implant placement protocol such as “dental implants”, “immediate placement”, “delayed/late placement” and terms related to healing processes following dental implant placement with search keywords including “osseointegration”, “implant wound healing”, “peri-implant bone healing”, and “foreign body reaction to biomaterials” were used to search and obtain data about the comparison of outcomes between immediate and delayed dental implant placement. Peer-reviewed in vitro, in vivo, clinical studies, and case reports published in English were included. These articles were reviewed and categorized based on the measurement strategies and reported outcomes to help identify differences between two placement protocols. As this is a scoping review, the included studies are heterogenous in various aspects such as study designs and observed parameters. Therefore, no further statistical analysis is conducted. Non-English publications were excluded from this review (Supplementary Materials).

## 3. Results

### 3.1. Timing of the Implant Placement—Terminologies & Classifications

In 1993, Wilson and Weber [11] classified treatment procedures for areas where implant cannot be placed due to bone insufficiency. The classification was based on the timing of tooth extraction in relation to implant placement and defined as follows:

- Immediate implant placement: same appointment as tooth extraction;
- Recent implant placement: 30–60 days after tooth extraction;
- Delayed implant placement: following hard tissue maturation;
- Mature implant placement: months to years after extraction.

However, this classification only addressed soft tissue healing and the predictability of guided bone regeneration, neglecting the time interval. Subsequently, Mayfield [12] proposed a classification that emphasizes the time interval for dental implant placement following tooth extraction. The terms “immediate”, “delayed”, and “late” were used to describe time intervals of 0 weeks, 6–10 weeks, and 6 months or more after extraction, respectively. Nevertheless, the interval between 10 weeks and 6 months was not addressed. Thereafter, Hämmerle and Lang [13] introduced implant placement protocols that were classified based on soft and hard tissue healing. This classification categorizes implant placement time into four types with a specific timeframe.

- Type 1: implant placement immediately following tooth extraction and as part of the same surgical procedure;
- Type 2: complete soft tissue coverage of the socket (typically 4 to 8 weeks);
- Type 3: substantial clinical and/or radiographic bone fill of the socket (typically 12 to 16 weeks);
- Type 4: healed site (typically more than 16 weeks).

**Figure 2:**
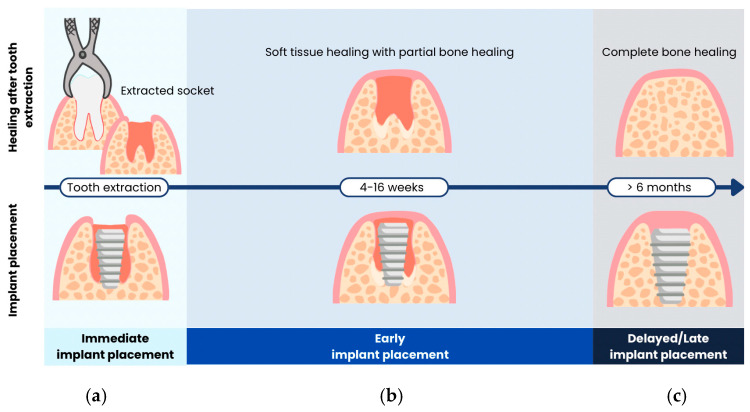
Different timeframes for implant placement following tooth extraction according to ITI 2018 [14], including (**a**) immediate, (**b**) early, and (**c**) delayed implant placement.

Most recently, a consensus statement was formulated by International Team for Implantology (ITI) in 2018 [14]. Implant placement protocols were defined as follows (Figure 2):

- Immediate implant placement: dental implants are placed in the socket on the same day as tooth extraction.
- Early implant placement: dental implants are placed with soft tissue healing (4–8 weeks) or with partial bone healing (12–16 weeks) after tooth extraction.
- Late or delayed implant placement: dental implants are placed after complete bone healing, more than 6 months after tooth extraction.

Numerous attempts have been made to classify the timing of implant placements. The data regarding definitions, advantages, and disadvantages associated with each placement time are summarized in Table 1.

According to the literature, DIP is a scientifically and clinically valid protocol. Nevertheless, a notable disadvantage of DIP is the risk of alveolar ridge bone resorption. This can result in insufficient bone volume, which may necessitate additional procedures such as bone grafting. Additionally, DIP often requires multiple operations and extended treatment time [11,12,13,14]. On the other hand, IIP has also been considered a predictable and acceptable procedure [3,5,6]. This approach offers several advantages, including a relatively shorter treatment duration and edentulous period, a reduced number of surgical procedures, maintenance of the soft tissue profile via customized healing abutments or immediate/early loaded provisional restorations, and optimization of available bone volume [15,16,17].

### 3.2. Comparison Between Immediate and Delayed Dental Implant Placement

The difference between IIP and DIP has sparked considerable scholarly investigation, resulting in a growing body of literature that attempts to understand the comparative advantages, drawbacks, and long-term outcomes of each protocol. The existing studies include in vivo preclinical studies, clinical studies, and systematic reviews and meta-analyses. The IIP and DIP protocols are often compared in terms of success and survival rates, periodontal status, esthetic outcome, radiographic examination, and biochemical analysis.

A total of 38 studies were included, comprising 6 pre-clinical studies and 32 clinical studies. The clinical studies were divided into 13 randomized controlled trials (RCTs) and 19 non-randomized studies (non-RCTs), including prospective cohort studies, retrospective studies, and case series. These studies primarily focused on implant placement in the anterior maxilla, though some extended to the posterior jaw and molar regions. The following section presents a detailed synthesis of findings across these studies, highlighting study designs and key differences between IIP and DIP.

#### 3.2.1. Pre-Clinical Studies

Pre-clinical studies on IIP mostly aim to evaluate the healing process by comparing histological changes over time to those observed in extraction socket [18,19,20] as well as to outcomes from DIP. Most of these studies are animal experiments, commonly conducted on beagle dogs [18,21,22,23] and mongrel dogs [24,25]. After receiving an experimental intervention, the animals were euthanatized, and specimens were obtained for histological analysis. The focused parameters included bone and soft tissue modeling, morphology, and bone-to-implant contact (BIC) [18,21,22,23,26].

Araújo et al. (2006) [18] conducted an experiment on 7 beagle dogs using the third and fourth premolars on both sides of the mandible to evaluate histological changes at day 0, week 4, and week 12 after IIP. On 5 dogs, implants were placed immediately after extraction on the right side of the jaw. Two months later, the same procedure was performed on the left side. All five dogs were then sacrified after 1 month of final implant placement, providing specimens that represented 4 and 12 weeks of healing. Another 2 dogs were used to represent 0 day after IIP. The implants were placed on both side of the animals’ jaw and the biopsies were taken within 2 h. Histological examination was performed under a microscope. Linear measurement was performed to access the distance between key landmarks, such as the implant shoulder, SLA surface, crest of the buccal or lingual bone wall, margin of the peri-implant mucosa, and apical termination of the barrier epithelium were identified [18].

Other studies used various experimental designs to compare between IIP and DIP. Schultes and Gaggl (2001) [23] divided experimental dogs into two groups; one received IIP and the other received DIP (6 months after extraction). The implants were left to allow osseointegration for 8 months and fluorochrome bone markers were injected twice before the dogs were sacrificed. Each implant and its surrounding tissue were examined for BIC and histology of hard and soft tissue [23]. In another study conducted by Sanz-Martin et al. (2017) [22], mesial roots of premolar and molar teeth were randomly assigned to the IIP or DIP using 2 implant designs (triangular at coronal half and cylindrical). The extraction sockets were left for two months to represent a healed site. After 8 weeks of healing, the same procedure was repeated at contralateral side of mandible. Four weeks after, samples were retrieved, thus providing two healing timepoints; 4 and 12 weeks. The healing of hard and soft tissues was evaluated using histology and micro-CT analysis. Soft tissue contour changes were assessed using image analysis software [22]. Similarly, Yi et al. (2017) [21] compared dimensional change of hard and soft tissues between extraction sockets, implant bed preparation, and the healing of implants utilizing IIP and DIP. All mandibular premolars were extracted and left to allow complete healing for 3 months. The left quadrant was used as the implant bed preparation site and M1 were extracted and used to represent alveolar socket healing. The right quadrant implants were placed at healed site to represent DIP. While, M1 were extracted and implants were placed immediately to represent IIP. After 8 weeks of bone healing, all samples were assessed histologically for new bone area/total area (BA/TA), BIC, and bone height changes from the buccal and lingual midlines [21]. On the other hand, Passoni et al. (2016) [25], designed a split-mouth experimental study on 7 mongrel dogs. Premolars were extracted and left for 120 days for healing, then implants were placed as per DIP. On the other side of the mandible at the time of DIP, premolars were extracted, and implants were placed immediately to represent IIP. After 4 months of osseointegration and 4 months post-loading, samples were collected and histomorphometric analysis was performed to determinate BIC [25]. The summary of the key findings of pre-clinical studies is shown in Table 2.

#### 3.2.2. Clinical Studies

The clinical research designs comparing the outcomes between IIP and DIP comprise retrospective studies, randomized controlled trials, and prospective cohort studies with a follow-up period of at least 6 months post-loading. In this study, we included 13 RCTs, 11 prospective cohort studies, 7 retrospective studies, and a case series. This study included single implant with proximal and occlusal contact in both maxilla and mandible.

When comparing clinical outcomes between IIP and DIP, the definition of IIP across studies is consistent, referring to implant placement following tooth extraction. Most studies excluded teeth with periapical lesions, with the exception of two studies that specifically investigated the success of implants in the presence of periapical lesions [28,29]. However, the definitions of healed ridge in DIP were not consistent among most previous studies. The timings for alveolar socket healing were varied from 2 months to more than 6 months, or even unspecified in some studies.

Across the numerous randomized controlled trials investigating IIP versus DIP, a broadly consistent protocol is apparent despite variations in follow-up duration, specific inclusion criteria, and adjunctive techniques (e.g., bone grafting, alveolar ridge preservation). Typically, each study recruits patients who need at least one tooth extraction in either the anterior or posterior jaw, then randomly allocates them to either have the implant placed immediately upon extraction or only after a specified healing period (usually 2 to 4 months). In some studies, alveolar ridge preservation was performed after tooth extraction in the DIP group [30,31,32,33,34,35,36]. In both groups, the implants are positioned according to prosthetic-driven principles. If necessary, grafting or augmentation is performed around any remaining bony defects. After surgery, patients generally receive standardized post-operative instructions, antibiotics, analgesics, and antiseptic mouth rinses. At follow-up appointments ranging from a few months to several years, investigators evaluate clinical indices (such as pocket depth, bleeding on probing, or keratinized mucosa), radiographic bone levels (e.g., marginal bone loss), esthetic parameters (commonly using a pink and white esthetic score, mid-buccal or papilla recession), and patient-reported outcomes on pain, satisfaction, or quality of life, as well as survival rate [28,29,30,31,32,33,34,35,36,37,38,39,40].

Given the operational difficulty and recruitment challenges that often limit sample size in randomized controlled trials, some investigators instead employ prospective cohort designs. This approach permits the enrollment of larger and more representative patient populations while still preserving the ability to observe temporal relationships between implant placement timing and subsequent clinical outcomes. Most of the recent non-randomized controlled studies performed in a single academic institution occasionally expanded to a small multicenter network. Patients who met the inclusion criteria were divided into IIP or DIP according to clinical circumstances, including the feasibility of IIP, the chronology of extraction, whether the patient presented with a failing tooth to be extracted or a healed ridge, or patient- or clinician-driven preference. The surgical, prosthetic, and follow-up protocols are closely aligned with those implemented in randomized controlled trials. Despite their heterogeneity in allocation methods, the non-randomized prospective cohorts measured a uniform set of endpoints, including radiographic bone level, buccal/palatal plate thickness, soft tissue thickness, esthetic indices, recording mid-facial recession, interdental papilla fill, modified plaque and gingival indices, probing depth, bleeding/suppuration, and keratinized-mucosa width. Finally, each study kept a record of events, documenting implant survival along with separate reports of biological complications (e.g., peri-implantitis) and technical issues (e.g., screw loosening, veneer chipping) [41,42,43,44,45,46,47,48,49,50].

For retrospective study design, data collection is primarily conducted through the review of existing patient records, including clinical notes and radiographs. This approach often allows for a longer follow-up period compared to other study designs, as it utilizes historical data. Once collected, the data are systematically categorized and analyzed to assess relevant clinical outcomes. Interestingly, a study conducted by Alexandre in 2023 [51] employed a split-mouth design and utilized data retrospectively gathered from patient records to evaluate clinical outcomes. This study employed a retrospective pilot design to evaluate and compare clinical, radiological, and patient-reported outcomes of IIP vs. DIP in individuals with tooth agenesis. The study included five non-smoking patients, all of whom presented with multiple agenesis sites. Utilizing a split-mouth approach, each patient received both IIP placed in extraction sites of retained deciduous teeth and DIP placed in previously healed edentulous sites. Data collection focused on implant survival, radiographic crestal bone level changes, and subjective outcomes including post-operative pain, patient satisfaction, and clinician-reported satisfaction. All implants were followed for at least one year post-placement. Notably, the study did not perform statistical analysis; rather, outcomes were reported descriptively. Ethical approval was deemed unnecessary due to the retrospective nature of the study and the limited sample size; however, all participants provided written informed consent [51].

#### 3.2.3. Treatment Outcomes and Their Assessments

##### Success and Survival Rates

Currently, there are no universally agreed-upon evaluation criteria for implant success. Several studies have examined the success and survival criteria for dental implants [4,5,6]. According to the Pisa Consensus Conference report from the International Congress of Oral Implantologists (ICOI), 7 survival criteria denote that the implant is retained in place rather than being removed from the mouth with no mobility, no pain from functions, or bone loss that exceeds half of the implant’s length [52]. Additionally, Albrektsson et al. defined that a successful implant must present no mobility, no peri-implant radiolucency, as well as no persistent pain, discomfort, or infection with bone loss less than 0.2 mm per year after the first year of loading. Failure of a dental implant is determined when an implant exhibits mobility, pain on functions, uncontrolled exudates, or severe bone loss [53].

Regarding implant survival rate, DIP demonstrates a range of 98.3–100%. IIP also exhibits a high survival rate, though with a slightly broader range of 83.7–100% [3,29,32,37,38,41,42,44,48,49,54,55,56]. Recent systematic reviews comparing IIP and DIP have raised a debate over whether IIP has a comparable survival rate to that of DIP. Some of them reported no significant difference in the survival rate of the implants [5,7,57], while others reported a significantly lower survival rate for IIP [4,58]. Nevertheless, these studies may not be directly comparable due to inconsistent study designs, as some included non-randomized control trials and multiple-unit implants, and the follow-up periods varied. Additionally, evaluations of survival rates were performed at different timepoints. Although earlier research did not show a statistically significant difference, several studies have reported a slightly lower survival rate for IIP [3,37,38,43,54,59]. A meta-analysis of randomized clinical trials conducted by Canellas et al. (2019) showed that the IIP had an increased risk of implant failure by 3% compared to DIP, which is in agreement with Ibrahim and Chrcanovic (2021) that reported a significantly higher risk of failure in IIP [60,61].

##### Periodontal Status

The parameters for evaluating peri-implant health and the extent of peri-implant diseases encompass various factors such as plaque assessment, mucosal conditions, peri-implant probing depth, the width of the peri-implant keratinized mucosa, peri-implant sulcus fluid, and radiographic evaluation [62].

Peri-implant probing depth (PD) is a frequently used metric for comparing clinical outcomes between IIP and DIP. The majority of prior studies agree that there is no statistically significant difference in peri-implant probing depths between the two implant placement protocols [4,6,29,32,37,47,54,63]. Only one study reported a trend of greater probing depths in IIP [38]. The increased probing depth in IIP can be attributed to the implant’s deeper placement, as IIP requires apical bone engagement during implant placement. However, limited information is available regarding whether these variations in biological healing and clinical parameters affect the local microbiome at dental implants, as well as the health and stability of the soft tissue seal.

Bleeding on probing (BOP) is a clinical indicator used to assess the health of the tissues surrounding dental implants. Its presence suggests inflammation [64]. This parameter was also reported in the studies comparing IIP and DIP. Cucchi et al. (2017) and Cooper et al. (2014) reported low bleeding scores below 15% for both IIP and DIP [37,41]. Conversely, Raes et al. (2018a) and Tonetti et al. (2017) showed high bleeding scores of over 30% in IIP [38,43]. Although there were variations in bleeding on probing scores, all studies agree that no statistically significant differences were observed [29,32,37,38,47,63].

Lastly, the width of keratinized mucosa is a frequently examined parameter in studies on dental implants, although only a limited number have specifically focused on comparisons between IIP and DIP [37,38,47,65]. This measurement is typically performed using a periodontal probe, with the distance measured from the restoration margin to the mucogingival junction. The majority of studies reported a stable band of keratinized tissue forming before implant placement throughout the follow-up period, with no significant difference between IIP and DIP [37,38,65]. On the contrary, Parvini et al. (2022) [47] proposed that the average width of buccal keratinized mucosa was significantly greater in IIP compared to DIP after 12 months of loading. Notably, in the IIP group, the width of the keratinized mucosa increased over time, from 6 months to 12 months of loading, whereas the DIP group exhibited a stable width of keratinized mucosa throughout the same period [47].

##### Esthetic Outcomes

Recent studies suggest that the esthetic outcomes are often evaluated by esthetic indices and positional changes of the peri-implant mucosa.

The gingival or papillary recession has been a frequently used criterion to evaluate the esthetic outcomes of IIP. However, the measurement presented heterogeneity across the studies, especially the recession measurement. The recession measurement could be categorized into:

- Direct periodontal probe readings: distance from reference points recorded with a UNC-15 or CP-15 probe (for mid-buccal recession: record the distance from the implant crown margin or CEJ of the contralateral/adjacent teeth to the mid-facial mucosal zenith, for the papilla recession: record the distance from the papilla tip to the contact point/incisal edge of adjacent teeth) [41,44,48,49,50].
- Calibrated photo-morphometry: standardized perpendicular intra-oral photographs were performed in each follow-up visit, and then the linear recession was calculated with image-analysis software against a calibration marker on the crown [33,42,46].
- Jemt papilla score: the classification described by Jemt [66]. These ranged from 0–4, representing (in order) 0 = no papillae, 1 = less than one-half of the gingival embrasure, 2 = at least one-half of the height, 3 = complete closure of the proximal space, and 4 = overgrowth.
- 3D surface superimposition: serial intra-oral scans file aligned on stable palatal reference by software and measured the change in soft tissue volume [47].

Most studies have shown no significant difference in the vertical change of papilla height following IIP and DIP [28,32,35,41,42,44,67], except for a study conducted by Raj et al. which demonstrated a statistically higher occurrence of papillary recession in IIP [50]. However, Raj et al.’s study utilized the Jemt papilla index as a measurement parameter, while other studies applied more precise methods, measuring the distance between reference points such as the crestal bone to the papilla crest, or the papilla crest to the contact point. Furthermore, the height of the papilla is primarily influenced by factors such as the gingival biotypes, the distance between the contact points of the crown, the level of the crestal bone, the location of the implant, and the morphology of the interproximal space [68,69,70,71]. Previously mentioned investigations were unable to control these confounding factors.

In terms of gingival recession, soft tissue change was often measured at the mid-buccal area. Most studies included in this article comparing IIP and DIP agreed that there was no significant difference between the placement time and mid-buccal recession [32,33,34,37,44]. In contrast, Cosyn et al. (2023) [36] found a significantly greater mid-facial recession in DIP compared to IIP, and suggested that DIP exhibited a substantial increase in soft-tissue loss in both horizontal and vertical measurements. Hence, it is challenging to attribute the variance solely to the timing of implant placement. A study suggests that it is possible to preserve soft tissue height and contour with an implant-supported temporary crown, which can be achieved after IIP [36].

In addition, Parvini et al. (2022) [47] proposed a novel approach to evaluate the alteration of soft tissues following an implant placement. An intraoral scanner was used to assess a 3D volumetric change of peri-implant tissues comparing IIP and DIP. The study reported that IIP in the esthetic region experienced more tissue loss compared to DIP [47].

The pink esthetic score (PES) is another commonly used index for a reproducible evaluation of the esthetic appearance of the soft tissues around crowns on single implants. It assigns seven points for the mesial and distal papilla, soft-tissue level, soft-tissue contour, soft-tissue color, soft-tissue texture, and alveolar process deficiency. The evaluation is carried out by visualizing papilla formation and comparing it to that of reference teeth [72]. Most of the current studies indicate that there is no significant difference in PESs between IIP and DIP [30,31,32,33,34,44,63,65,73]. Only a few literature reported opposite findings; Tonetti et al. (2017) reported PESs favoring IIP [38]. In contrast, Ghahroudi et al. (2020) reported better PESs in DIP [46].

##### Radiographic Examination

The standard methods for evaluating the change in bone volume after implant placement include periapical radiographs with a parallel technique using an extension cone parallelled (XCP) combined with a customized occlusal jig, as well as cone beam computed tomography (CBCT). Many studies often employ marginal bone loss (MBL) as their measuring approach. MBL can be assessed using both 2D periapical radiographs and CBCT. However, reproducing parallel radiographs using the periapical technique, even with a customized jig, remains challenging. Therefore, the accuracy of this method is largely dependent on various factors. In contrast, CBCT may offer greater accuracy in assessing MBL. Previous studies indicate that there is no significant difference in MBL at least one year post-loading between the two placement protocols. [4,6,29,34,41,43,48,49,54,55,61,74]. Some other studies showed conflicting results in which DIP appeared to have less MBL [32,38,39,50]. The observed difference may be attributed to the risk of bias arising from broad inclusion criteria that did not exclude sockets with buccal wall defects in the IIP group. Notably, these studies assessed MBL using periapical radiographs.

On the other hand, only a few studies investigated the change in horizontal bone thickness using CBCT, and the findings have remained controversial [29,30,31,32,33,35,40,44,45,48].

##### Histological Examination

From a histological point of view, at 4 weeks after IIP, new bone had filled the gap between the implant and socket, forming contact with the implant surface, though early resorption of the buccal and lingual walls was noted. By 12 weeks, buccal bone resorption had progressed further, leading to apical migration of the buccal crest, while the lingual bone height remained relatively stable [18]. This shows that IIP does not prevent the remodeling and subsequent bone loss that naturally occurs after a tooth extraction [18,19,21,75]. Bone healing around IIP primarily relies on the distance osteogenesis rather than contact osteogenesis [76]. In terms of BIC, IIP presents better BIC scores compared to DIP [21,22,25]. Additionally, IIP appears to have a formation of a longer soft tissue attachment at the cervical region, resulting from crestal bone resorption after implant placement [20]. Regarding soft tissue, in both placement procedures, the peri-implant mucosa was covered with a keratinized oral epithelium adjacent to a barrier epithelium and facing a polished surface of the implant abutment after healing of soft tissues. Physiologically, the connective tissue areas exhibit well-organized collagen fiber bundles and vascular structures [18].

##### Biochemical Analysis

Alongside standard clinical examinations, several studies have focused on molecular dynamics of the bone and soft tissue surrounding implants with an emphasis on the process of osseointegration [77,78,79]. These studies aim to investigate changes in biomarkers and molecular markers to explore their potential association with clinical outcomes. Biomarkers are biochemical indicators that reflect actual or potential changes in the structure and function of cells and tissues. These biomarkers range from genes, RNAs, proteins, and metabolites, and play important roles in disease diagnosis, prognosis, prediction, and treatment [80]. Various techniques have been used to analyze the molecular activities. PICF is a site-specific and easily collectible biofluid that could be subjected to biochemical analysis of immunological biomarkers by a noninvasive method [81]. The fluid contains biomarkers, such as cytokines, proteins, and multifunctional peptides, which modulate inflammation intensity, foreign body reaction, cellular organization, healing processes, and disease pathogenesis [82,83].

Gürkan et al. (2019) [79] reported changes in cytokines during healing after implant placement with the peak of Interleukin-1β (IL-1β), granulocyte colony-stimulating factor (G-CSF), IL-13, IL-6, IL-12, interferon-γ (IFN-γ), IFN-α, IL-2, IL-2R, IL-8, macrophage inflammatory protein (MIP)-1α, MIP-1β, monocyte chemoattractant protein-1 (MCP-1), interferon gamma-induced protein (IP)-10, monokine induced by IFN-γ (MIG), epidermal growth factor (EGF), hepatocyte growth factor (HGF), and vascular endothelial growth factor (VEGF) at week 2 after the implant placement, indicating an acute reaction to surgical trauma. The response showed a notable decline over time by week 4 to week 8. These biological findings suggest that the initial phase of peri-implant wound healing is completed between the second to twelveth week after surgery [79]. Similarly, Bielemann et al. (2018) [77] reported high levels of proinflammatory cytokines such as ILs, tumor necrosis factor (TNF), and IFN-γ along with alkaline phosphatase (ALP), matrix metalloproteinase-1 (MMP-1), MMP-8, and collagenase activity during the first week of healing. On the other hand, IL-10, an anti-inflammatory cytokine, shows a progressive increase during week 1 to week 12 of bone healing, suggesting its role in resolving inflammation and regulating bone homeostasis [77]. During the late healing phase (8 weeks after implant placement), biomarkers such as IL-1β, TNF-α, Cathepsin K (CatK), and Tartrate-resistant acid phosphatase (TRAP), which are associated with osteoclastogenesis and bone resorption, can be observed. IL-1β has been correlated with complications like bone dehiscence and implant instability. During this phase, IL-1β levels decrease, and bone apposition is directed towards greater activity [77]. However, quantifying osseointegration biomarkers and determining their phase-specific significance was not possible in this referenced systematic review.

Current knowledge is largely limited to identifying biomarkers involved in each phase of osseointegration, while their direct relationship to clinical outcomes remains unclear. Moreover, there is no available literature comparing biomarker expression during the healing process between IIP and DIP. From a biological standpoint, it is still uncertain whether the healing processes in IIP and DIP follow similar or distinct pathways.

Genomic analysis is a less common method for studying the molecular-level differences in implantology. However, the development of genomics and epigenomics may potentially help identify individual susceptibility and, hence, have been utilized to study multifactorial and complex diseases. Small noncoding RNAs known as microRNAs (miRNAs) control the expression of genes by binding to complementary sequences in the 3′-untranslated or coding sections of target mRNAs, which silences the corresponding gene. They play a crucial role in controlling the inflammatory and immunological responses of the host to pathogens [84]. Previous studies have shown that a considerable number of microRNAs can be detected in peri-implant crevicular fluid and peri-implant tissues [85,86]. Slotte et al. (2012) [85] investigated gene expression of inflammation and bone healing in PICF after placement and loading of dental implants and suggested that the gene expressions for IL-1β, TNF-α, and ALP were much higher compared to osteocalcin (OCN), cathepsin K, and tartrate resistant acid phosphatase (TRAP). Moreover, IL-1β, TNF-α, and ALP are more actively involved in the healing and inflammatory processes. TNF-α was initially highly expressed and then gradually decreased over time and showed correlation with clinical complications (ie, bone dehiscence at the implant surface at surgery, rotation instability of implant, loose implant or implant removal) at days 2 and 14. On the other hand, OC and ALP showed a slight increase in expression over time but only ALP showed a correlation with clinical parameters such as the wound healing index (WHI), resonance frequency analysis (RFA), and clinical complications. Interestingly, there was no clear pattern or significant changes in the spatial distribution of IL-1β, ALP, CK, and TRAP gene expressions. Lastly, the correlations between gene expression and clinical parameters were observed at different timepoints. Of these, WHI, RFA and clinical complications displayed more correlations with gene expression than the other parameters and showed significant correlations with ALP, TNF-α, and IL-1β [85].

**Figure 3:**
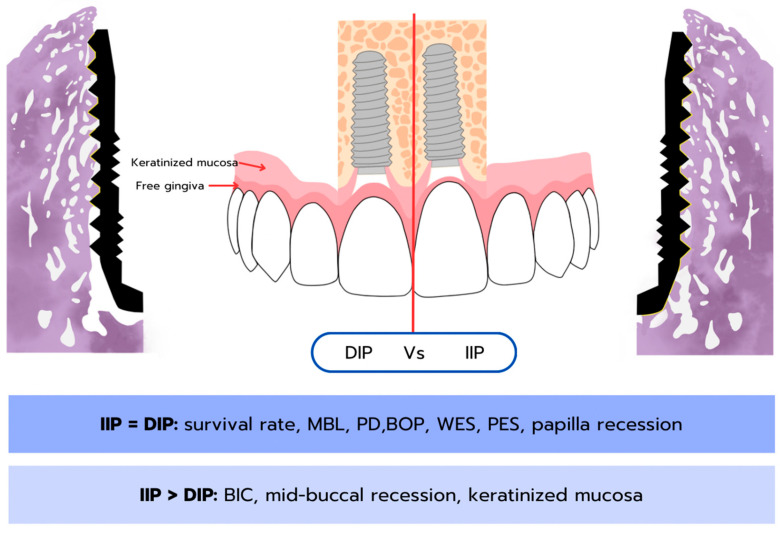
The significantly different and comparable parameters regarding the outcomes of IIP and DIP (MBL; marginal bone loss, PD; pocket depth, BOP; bleeding on probing, WES; white esthetic score, PES; pink esthetic score, and BIC; bone to implant contact).

In conclusion, various study designs and numerous parameters have been utilized to compare the outcomes of IIP and DIP biologically and clinically in vitro, in vivo, and in clinical studies. Despite the heterogeneity of previous studies, most parameters remain comparable in terms of implant placement outcomes, while some observed parameters show significant differences between IIP and DIP. The summary of the key findings of these studies is shown in Table 3 and Table 4 and Figure 3.

## 4. Discussion

Our review has highlighted the similarities and differences between IIP and DIP in various aspects, including treatment outcomes, as well as the heterogeneity among studies to date. Firstly, several attempts have been made to categorize the timing of implant placement. The most commonly recognized terms for implant placement protocol are immediate, early, and delayed implant placement. However, the definitions of these timeframes vary across different classification systems, often leading to overlapping timelines. As a result, interpreting studies with different terminologies of implant placement timing can be challenging, making it difficult to accurately compare or consolidate findings from different articles. More recent studies have attempted to avoid this confusion by adopting a more time-specific classification, namely the ITI Consensus Statement of 2004, which categorizes implant placement timing into four distinct types [13].

- Type I (immediate implant placement): immediately after tooth extraction;
- Type II (early implant placement with soft tissue healing): 4–8 weeks after tooth extraction;
- Type III (early implant placement with partial bone healing): 12–16 weeks after tooth extraction;
- Type IV (delayed implants with soft and hard tissue healing): 4 months after tooth extraction.

Secondly, numerous studies on IIP have encompassed both pre-clinical and clinical investigations over the past decades. In vivo studies are predominantly conducted in beagle dogs, primarily to investigate the biology of wound healing following implant placement through histological analyses. Researchers attempted to monitor the histological change throughout process of osseointegration, while the main focus was typically dimensional change of surrounding bone around an implant. However, only a few studies explore cellular behavior around IIP. A study by Watanabe et al. (2016) [27] suggested that granulation tissue and bone formation progressed more quickly in IIP in the early healing stage (Day 5), along with increased presence of TRAP-positive cells, compared to DIP. By Day 14, cell proliferation was significantly higher in the immediate group, further supporting the idea that immediate placement may offer advantages in early tissue healing [27]. These findings suggest that remnants of periodontal tissue in the extraction socket may support early healing around IIP, especially in cases where there is a gap between the socket wall and the implant [27,93]. In DIP, the surrounding bone often undergoes high compressive strain during implant placement, leading to osteocyte death and subsequent bone resorption. In contrast, IIP tends to occur in areas of lower mechanical strain, resulting in less osteocyte damage and more favorable peri-implant bone formation [93].

Nevertheless, these studies have limited applicability to human clinical practice due to species-specific physiological differences.

Consequently, the focus has shifted to clinical studies for comparing clinical outcomes of IIP and DIP. When success and survival rates are determined by implants remaining in the oral cavity, both placement protocols appear to yield comparable outcomes, similar to other clinical parameters such as MBL, PD, BOP, WES, and PES. These results are consistent with those of a previous systematic review, which reported no significant difference in implant survival rates, MBL, and soft tissue health between the two groups; however, the review noted that IIP demonstrated a slightly lower survival rate and slightly higher MBL compared to DIP [5,6,7,58,94]. In terms of MBL, the minority of studies included in this review also tended to favor DIP. A common feature of these studies is that they assessed MBL using periapical radiographs rather than CBCT and employed a delayed loading protocol. Moreover, these trials did not regulate the depth of implant placement in either group. MBL was often assessed from the crestal bone and implant platform reference point. Hence, the IIP was usually positioned further sub-crestally to counteract alveolar bone remodeling [32,38,39,50]. Therefore, loading time and implant position are important factors to consider when interpreting results related to MBL. Systematic reviews by Pommer et al. (2021) suggested that MBL was statistically lower or comparable in immediate loading when compared to delayed or conventional loading, given that appropriate surgical protocols were followed [94].

Peri-implant soft tissues and esthetic indices are utilized as esthetic parameters. Although this study clearly shows no difference in PES, WES, and soft tissue recession, the results of this study are not consistent with the findings of the systematic review that included a comparison of IIP, early implant placement (EIP), and DIP. The systematic reviews by Chen & Buser (2014), Cosyn et al. (2019), and Bassir et al. (2019) suggested that IIP had a higher incidence of mid-buccal recession when compared to EIP and DIP [4,6,95]. These findings align with the 2008 ITI consensus statement, which identified IIP as a risk factor for mucosal recession [94,95,96]. The inconsistency may arise from the included studies adhered to a strict protocol for IIP, which involved atraumatic tooth extraction, a flapless procedure, preservation of the buccal bone wall with a minimum thickness of 1 mm., and the absence of soft tissue grafting. Similarly, when examining studies that focus solely on IIP, it is evident that IIP is associated with a higher frequency of recession. Systematic reviews by Cosyn et al. (2012) and Wu et al. (2023) reported midfacial soft tissue recession as a common issue, with advanced recession (greater than 1 mm) occurring in 0–53% of cases [97,98]. Cosyn et al. suggested that factors contributing to increased recession in IIP include a thin gingival biotype and the lack of immediate crown restoration. The study further indicated that an intact buccal bone wall and a thick gingival biotype may help reduce the risk of significant recession [97]. Notably, the studies comparing immediate and delayed implant placement (IIP and DIP) included in this review did not assess the effects of different loading protocols on clinical outcomes. Both immediate and delayed loading protocols were represented among the included studies, but their impact was not separately analyzed. Similarly, the influence of bone grafting around IIP or DIP was not evaluated. Due to the heterogeneity among the studies, we can only identify general trends in clinical outcomes between the two placement protocols. Therefore, the results cannot determine the relevance of other factors that may influence outcomes in IIP.

Despite the heterogeneity among the studies, they suggest that the amount of bone at the implantation site is likely to play a role in clinical outcomes of IIP. Post-extraction buccal bone thickness plays a pivotal role in the predictability of IIP, as the buccal plate is highly susceptible to rapid resorption following tooth extraction [18,19]. An intact buccal bone wall, preferably with a minimum thickness of approximately 1 mm, is considered critical to minimizing horizontal and vertical bone loss and reducing the risk of mid-facial soft-tissue recession, particularly in esthetic regions [97]. Following IIP, a “jumping gap” often exists between the implant surface and the surrounding bony socket; histological evidence indicates that this gap can undergo spontaneous healing through distance osteogenesis even in the absence of grafting, provided that the gap is limited, and the buccal plate remains intact [76]. However, in cases where the jumping gap is wide (>2 mm), or the buccal bone is thin or compromised, the use of bone grafting may be beneficial to support ridge contour, limit resorption, and enhance peri-implant tissue stability [99]. Although clinical evidence suggests that IIP may achieve acceptable outcomes with or without grafting under favorable conditions, adjunctive grafting appears particularly advantageous in esthetic sites where preservation of buccal bone thickness and soft-tissue architecture is critical for long-term esthetic success. However, the use of specific bone grafting material over another for the best outcomes of IIP has remained inconclusive, although the xenogeneic graft from bovine origin is frequently used [100].

The importance of the macro- and microtopography of implants on treatment outcomes irrespective of the placement protocols has been well-established. However, in the present review, implant size and macrodesign varied substantially among the retrieved in vivo studies due to differences in animal models and experimental setups, limiting their direct relevance to commercially available implants used in humans. On the other hand, thread as a macrotopography has been mostly utilized in clinical studies. Regarding the micro- and nanotopography which is the result of surface modifications, sandblasting and acid etching (commonly referred to sandblasted large-grit acid-etching; SLA) have been mostly used to establish various scales of surface roughness, while surface treatment protocols differ among manufacturers and are not consistently reported across studies. Interestingly, the correlation between implant designs and treatment outcomes of IIP and DIP has not been reported and compared. Nevertheless, currently available implant designs on the market, particularly threaded implants with SLA or comparable surface modifications, generally demonstrate high survival and success rates in healthy patients with adequate bone density. In clinical situations involving low bone density, and likely in IIP scenarios, implant features such as a conical titanium design, wider diameter, increased length, reverse buttress threads with self-tapping capability, smaller thread pitch, and deeper thread depth may potentially enhance primary stability and improve treatment outcomes [101].

While the primary focus of the present review is the placement protocols, it is also important to address loading protocols as a complementary factor influencing treatment outcomes. Immediate loading has become an increasingly common adjunct to immediate implant placement, particularly in partially edentulous and full-arch rehabilitations, where the number of implants subjected to functional load plays a critical role in load distribution and peri-implant tissue response. A recent systematic review evaluating immediately loaded implant-supported fixed partial prostheses in posterior regions reported favorable survival rates ranging from 86% to 100%, with follow-up periods extending up to 10 years, despite notable heterogeneity in implant designs, prosthetic materials, and loading protocols. These findings suggest that, under appropriate clinical conditions and with adequate primary stability, immediate loading may represent a reliable and patient-centered treatment option. Nevertheless, the variability in study designs and loading strategies highlights the need for careful case selection and underscores the importance of considering biomechanical factors, such as the number and distribution of implants, when extrapolating immediate loading outcomes to IIP scenarios [102].

To better understand the biology of osseointegration in human post-extraction sockets, Nevins et al. (2018) [99] conducted a human histological study. Five implants were retrieved using an “en bloc” biopsy technique for histological analysis after six months of healing, confirming successful osseointegration around IIP [99]. However, this approach provides no insight into the early stages of healing. Since time-lapse histological studies in humans are nearly impossible to perform, the use of biomarkers has emerged as a valuable, non-invasive tool for monitoring the dynamic healing process and detecting biological change. These quantifiable indicators play a role in identifying early signs of pathological conditions [103]. In implant dentistry, biomarkers were used to evaluate peri-implant tissue responses, including inflammation, bone remodeling, and healing. Previous studies suggested that changes in biomarker levels such as collagenase-2,3, ALP, IL-1β, IL-6, IL-17, TNF-α, osteoprogeterin (OPG), and soluble receptor activator of nuclear factor kappaB ligand (sRANKL) could predict peri-implant bone loss [104].

To date, no studies have directly investigated differences in biomarker expression between IIP and DIP. Most existing research has focused on comparing biomarker levels in relation to different loading protocols rather than placement protocols. Among the most commonly studied biomarkers are OCN and TNF-α, while clinical parameters such as peri-implant sulcular depth, bleeding and gingival index are frequently assessed alongside biomarker data. However, these studies have not demonstrated a strong correlation between the examined biomarkers and clinical outcomes [78,89,90,91,92]. For example, elevated levels of TNF-α during early healing phase (days 2–14) had been associated with clinical complications [78], while OCN is typically linked to the later stages of bone formation [89].

Across studies with comparable results between IIP and DIP, consistent surgical protocols and patient selection profiles were observed. This suggests that, with appropriate patient selection and adherence to a standardized surgical protocol, IIP can yield promising outcomes. Patients receiving IIP should be systemically healthy, non-smoking adults who can maintain full-mouth plaque and bleeding scores of less than 20% [34,36,54]. The failing tooth should be atraumatically extractable with an intact buccal socket wall [34,36,54,63,88]. A thick gingival biotype is strongly preferred, as thin scalloped biotypes are consistently associated with facial recession [88]. Acute suppuration is an absolute exclusion criterion. Presence of periapical lesion is not a contraindication for IIP, proper debridement and antibiotic coverage should be performed [28,29]. Lastly, radiographic analysis must confirm at least 3 mm of apical and palatal bone for implant stability, with a minimum primary stability of 25–30 Ncm [14].

Interestingly, we found that a notable limitation in recent research lies in the relatively small number of subjects and the brief duration of follow-up periods. Future studies should address this limitation by considering a larger subject pool and extending the prospective follow-up period to enable a more robust statistical comparison between groups. Most of the studies on IIP and DIP were conducted on different individuals, and interpersonal differences cannot be fully controlled. An ideal study design would involve split-mouth randomized clinical trials to minimize these variables. Beyond current clinical knowledge with various limitations of studies shown in Table 5, it remains important to understand the biological differences in the healing processes between IIP and DIP. The biology of wound healing difference between placement protocols has remained a crucial knowledge gap in the field. At present, clinical outcomes largely depend on the surgeon’s skill and appropriate case selection. Gaining deeper insight into the underlying biology could support the development of biomarkers as indicators for better case selection, predicting treatment outcomes and handling complications, or even lead to molecular strategies that enhance the clinical success of IIP in restoring missing teeth. Therefore, future research endeavors should focus on investigating the biological aspects of wound healing between IIP and DIP using well-controlled study designs, such as split-mouth randomized controlled trials.

## 5. Conclusions

The debate between IIP and DIP revolves around various factors including bone quality, soft tissue condition, and patient preferences. IIP offers the advantage of reducing treatment time by eliminating the need for a second surgical procedure, preserving esthetics, and reducing edentulous period, thereby enhancing patient satisfaction. Many pre-clinical and clinical models, along with various parameters and measurement techniques, have been utilized throughout these past decades. From a biological point of view, IIP demonstrates greater BIC compared to DIP. With regard to clinical outcomes, IIP demonstrates no significant differences in terms of success rate, survival rate, MBL, PD, BOP, WES, PES, and recession. It is important to note that many studies suggest IIP is associated with a risk of mid-buccal recession, which could compromise esthetic outcomes in the future. However, it is suggested that IIP is an effective treatment protocol, yielding results comparable to those of DIP. In healthy, non-smoking patients with unrestorable teeth that can be extracted while preserving the buccal bone, IIP can be performed following a strict surgical protocol, leading to promising clinical outcomes. Furthermore, recent studies on IIP have limitations regarding small sample sizes, short follow-up periods, and interpersonal variations, necessitating larger sample pools, long-term investigations, and a split-mouth design trial for more reliable comparison. Lastly, there is a knowledge gap in understanding the biological differences in wound healing following IIP and DIP, which should be further investigated to improve treatment outcome predictions and complication managements in both protocols.

## Figures and Tables

**Table 1:** Classifications of implant placement protocols and their advantages and disadvantages [11,13,14].

Classification		Placement Time	Advantages	Disadvantages
Name	Author			
Immediate implant placement	Wilson & Weber [11]	Immediately following tooth extraction and as part of the same surgical procedure	Reduced number of surgical procedures Reduced overall treatment time Optimal availability of existing bone	Site morphology may complicate optimal placement and anchorage Thin tissue biotype may compromise optimal outcomes Potential lack of keratinized mucosa for flap adaptation Adjunctive surgical procedures may be required Procedure is technique-sensitive
	ITI [14]			
Type I	Hämmerle [13]			
Recent implant placement	Wilson & Weber [11]	Complete soft tissue coverage of the socket (typically 4–8 weeks)	Increased soft tissue area and volume facilitates soft tissue flap management Resolution of local pathology can be assessed	Site morphology may complicate optimal placement and anchorage Treatment time is increased Socket walls exhibit varying amounts of resorption Adjunctive surgical procedures may be required Procedure is technique-sensitive
Early implant placement with soft tissue healing	ITI [14]			
Type 2	Hämmerle [13]			
Delayed implant placement	Wilson & Weber [11]	Substantial clinical and/or radiographic bone fill of the socket (typically 12–16 week)	Substantial bone fill of the socket facilitates implant placement Mature soft tissues facilitate flap management	Treatment time is increased Adjunctive surgical procedures may be required Socket walls exhibit varying amounts of resorption
Early implant placement with partial bone healing	ITI [14]			
Type 3	Hämmerle [13]			
Mature implant placement	Wilson & Weber [13]	Completely healed extraction site (typically more than 16 weeks)	Clinically healed ridge ensures optimal healing Mature soft tissues facilitate flap management	Treatment time is increased Adjunctive surgical procedures may be required Large variations are present in available bone volume
Late or delayed implant placement	ITI [14]			
Type 4	Hämmerle [13]			

**Table 2:** The summary of the key findings of pre-clinical studies regarding the outcomes of IIP and DIP.

Authors & Year	Healing Period	Histological Outcomes		
		BIC	Crestal Bone Loss	Healing Processes & Soft Tissues
Schultes & Gaggl, 2001 [23]	8 months	IIP = DIP	N/A	DIP > IIP Denser fibrous tissues More hemidesmosomes
Watanabe et al., 2016 [27]	4 weeks	IIP = DIP	IIP = DIP	IIP > DIP
Passoni et al., 2016 [25]	4 months	IIP > DIP	N/A	N/A
Sanz-Martin et al., 2017 [22]	4 and 12 weeks	IIP > DIP (at 12 weeks)	N/A	IIP = DIP Soft tissue dimension and contour Hard and soft tissue integration
Yi et al., 2017 [21]	8 weeks	IIP > DIP	IIP > DIP	N/A
Lee et al., 2022 [24]	3 months	N/A	IIP > DIP (buccal bone)	IIP = DIP = EIP Ridge width shrinkage Ridge profile

**Table 3:** The summary of the key findings of clinical studies regarding the outcomes of IIP and DIP (Ant; anterior, Post; posterior region, Max; maxilla).

Author & Year	Study Design	Area	Loading Protocol	Healing Period	Follow-Up Period	Outcomes							
						Survival Rate	MBL	PD	BOP	Keratinized Mucosa	Recession	PES	WES
Lindeboom et. al., 2006 [28]	RCT	Ant Max	Delayed	6 months	1 year	N/A	IIP = DIP	N/A	N/A	N/A	IIP = DIP (papilla)	N/A	N/A
Mangano et al., 2013 [73]	Retrospective study	Ant Max	Immediate	3 months	31.09–34.44 months	N/A	N/A	N/A	N/A	N/A	N/A	IIP = DIP	IIP = DIP
Cooper et al., 2014 [41]	Retrospective study	Ant Max	Immediate	11–12 weeks	5 years	IIP = DIP	IIP = DIP	N/A	N/A	N/A	IIP = DIP (papilla)	N/A	N/A
Hof et al., 2015 [65]	Retrospective study	Ant Max	N/A	N/A	51.2 months	N/A	IIP = DIP	IIP = DIP	IIP = DIP	IIP = DIP	IIP = DIP	IIP = DIP	IIP = DIP
Cucchi et al., 2017 [37]	RCT	Post	Delayed	3 months	1–3 years	IIP = DIP	IIP = DIP	IIP = DIP	IIP = DIP	IIP = DIP	IIP = DIP	N/A	N/A
Tonetti et al., 2017 [38]	RCT	Ant Max	Delayed	12 weeks	1–3 years	N/A	IIP > DIP (at 36 months)	IIP > DIP	IIP = DIP	IIP = DIP	N/A	IIP > DIP	IIP = DIP
Kniha et al., 2017 [42]	RCT	N/A	Immediate	3 months	1 year	IIP = DIP	N/A	N/A	N/A	N/A	IIP = DIP (papilla)	N/A	N/A
Raes et al., 2018a [43]	Prospective cohort	Ant Max	Immediate	10 weeks	8 years	N/A	N/A	N/A	N/A	N/A	N/A	IIP = DIP	N/A
Raes et al., 2018b [44]	Prospective cohort	Ant Max	Immediate	10 weeks	8 years	N/A	IIP = DIP	N/A	N/A	N/A	N/A	N/A	N/A
Pour et al., 2018 [63]	Retrospective study	N/A	Delayed	6 months	14.42–18.25 months	N/A	IIP = DIP	IIP = DIP	IIP = DIP	N/A	N/A	IIP = DIP	N/A
Kato et al., 2018 [45]	Prospective cohort	Ant Max	Delayed	N/A	1 year	IIP = DIP	IIP = DIP	N/A	N/A	N/A	N/A	N/A	N/A
Ghahroudi et al., 2020 [46]	Prospective cohort	Ant Max	N/A	N/A	35 months	N/A	N/A	N/A	N/A	N/A	N/A	DIP > EIP, IIP	N/A
Singh et al., 2021 [39]	RCT	N/A	Delayed	N/A	3 and 6 months	N/A	IIP > DIP	N/A	N/A	N/A	N/A	N/A	N/A
Santhanakrishnan et al., 2021a [30]	RCT	Ant Max	Immediate	3–4 months	6 months	N/A	N/A	N/A	N/A	N/A	N/A	IIP = DIP	N/A
Santhanakrishnan et al., 2021b [31]	RCT	Ant Max	Immediate	4 months	6 months	N/A	N/A	N/A	N/A	N/A	N/A	IIP = DIP	N/A
Slagter et al., 2021 [32]	RCT	Ant Max	Delayed	3–6 months	5 years	IIP = DIP	IIP > DIP	IIP = DIP	IIP = DIP	N/A	IIP = DIP	IIP = DIP	N/A
Parvini et al., 2022 [47]	Prospective cohort	Ant	Immediate	3–6 months	6 and 12 months	N/A	N/A	IIP = DIP	IIP = DIP	IIP > DIP (at 12 months)	IIP = DIP	N/A	N/A
Chatzopoulos & Wolff, 2022 [3]	Prospective cohort	All	N/A	N/A	68.9–75.1 months	IIP = DIP	N/A	N/A	N/A	N/A	N/A	N/A	N/A
Al-Aroomi et al., 2023 [55]	Prospective cohort	All	Delayed	N/A	19.2–19.5 months	IIP = DIP	IIP = DIP	N/A	N/A	N/A	N/A	N/A	N/A
Cardaropoli et al., 2022b [35]	RCT	Ant Max	N/A	6 weeks	1 year	N/A	N/A	N/A	N/A	N/A	IIP = DIP	IIP = DIP	N/A
Cardaropoli et al., 2022a [34]	RCT	Ant Max	N/A	6 weeks	1 year	N/A	IIP = DIP	N/A	N/A	N/A	N/A	N/A	N/A
Dagher et al., 2022 [48]	Prospective cohort	Premolar Max	Immediate	N/A	6 months	IIP = DIP	IIP = DIP	N/A	N/A	N/A	N/A	N/A	N/A
Zamparini et al., 2023 [74]	Prospective cohort	N/A	Delayed	3 months	10 years	N/A	IIP = DIP (at 10 years)	N/A	N/A	N/A	N/A	N/A	N/A
Carosi et al., 2023 [54]	Case series	Ant	Immediate	6–12 weeks	1 year	N/A	IIP = DIP	N/A	N/A	N/A	N/A	N/A	N/A
Kumar et al., 2023 [49]	Prospective cohort	N/A	N/A	N/A	1, 3 and 6 months	IIP = DIP	IIP = DIP	N/A	N/A	N/A	N/A	N/A	N/A
Cosyn et al., 2023 [36]	RCT	Ant Max	Immediate	N/A	6 months	N/A	N/A	N/A	N/A	N/A	IIP < DIP (mid-facial)	N/A	N/A
Yu et al., 2023 [29]	RCT	Molar	Delayed	6 months	1 year	IIP = DIP	IIP = DIP	IIP = DIP	IIP = DIP	N/A	N/A	N/A	N/A
Raj et al., 2023 [50]	Prospective cohort	All	Delayed	N/A	6 and 12 months	N/A	IIP > DIP	N/A	N/A	N/A	IIP > DIP (papilla)	N/A	N/A
Alam et al., 2024 [87]	Retrospective study	N/A	N/A	N/A	5 years	IIP = DIP	IIP = DIP	N/A	N/A	N/A	N/A	N/A	N/A
Schiegnitz et al., 2024 [88]	Retrospective study	N/A	N/A	4 months	12 months	IIP = DIP	IIP = DIP	N/A	N/A	N/A	N/A	N/A	N/A
Ghazal et. al., 2024 [40]	RCT	N/A	N/A	N/A	12 months	N/A	IIP = DIP	N/A	N/A	N/A	N/A	N/A	NA
Meijer et al., 2025 [33]	RCT	Ant Max	Delayed	3 months	10 years	IIP = DIP	IIP = DIP	IIP = DIP	IIP = DIP	N/A	IIP = DIP	IIP = DIP	IIP = DIP

**Table 4:** The summary of comparative outcomes between IIP and DIP.

Outcomes	IIP = DIP (No Significant Difference)	IIP > DIP	DIP > IIP	Potentially-Related Biomarkers
Survival rate	13 studies [3,29,32,33,37,41,42,45,48,49,55,87,88]			
Marginal bone loss (MBL)	17 studies [28,29,33,35,37,40,41,43,45,48,49,54,55,63,65,87,88]	4 studies [32,38,39,50]		OCN, TNF-α, ALP, IL-1β [78,89,90,91,92]
Buccal bone thickness	2 studies [32,33,34,40,44,45]	4 studies [30]	2 studies [29,48]	
Pocket depth (PD)	7 studies [29,32,33,37,47,63,65]	1 study [38]		TNF-α, IL-1β [78]
BOP (bleeding on probing)	7 studies [29,32,33,37,38,47,65]			TNF-α, IL-1β [78]
Keratinized mucosa	3 studies [37,38,65]	1 study [47]		
Soft tissue recession	9 studies [28,32,33,34,37,41,42,44,47,65]		1 study [36]	
Pink esthetic score (PES)	9 studies [30,31,32,33,34,44,63,65,73]	1 study [38]	1 study [46]	
White esthetic score (WES)	4 studies [33,38,65,73]			
Complication rate (e.g., Wound failure)		1 study [38] (IIP has higher wound failure)		
Soft tissue volume loss	3 studies [34,44,45]	1 study [47]	1 study [36]	

**Table 5:** Limitations of the clinical trials included in this review regarding IIP vs. DIP in various aspects.

Limitations	Details
Study designs	Significant heterogeneity among included studies Pre-clinical studies [19,21,22,23,24,25,27] Clinical studies ○ RCTs [28,29,30,31,32,33,34,35,36,37,38,39,40] ○ Prospective cohort studies [41,42,43,44,45,46,47,48,49,50,74] ○ Retrospective studies [3,55,63,65,73,87,88] ○ Case-series [54]
Definitions of placement time	Among the included studies, the definition of DIP varied the most and defined as: Implant placed > 10 weeks post-extraction [43,44] Implant placed > 3 months post-extraction [3,28,32,33,38,41,42,87] Implant placed > 4 months post-extraction [30,31,34,35,36,37,47,63] Implant placed > 6 months post-extraction [29,49,55,63,65] Implant placed on healed ridge [54,73]
Loading protocols	The included studies combined both immediate and delayed loading protocols, without properly grouping them to separate the influence of loading from that of placement protocols. This lack of distinction may have affected the interpretation of clinical outcomes. Immediate loading [30,31,36,41,42,43,44,47,48,54,73] Delayed loading [28,29,32,33,37,38,39,45,50,55,63,74]
Surgeon’s skills and surgical techniques	The included studies exhibited methodological variability, incorporating different surgical techniques such as flap and flapless procedures, the use of bone grafting, and data collected from multiple clinical centers.
Follow-up timepoints and durations	The included studies showed inconsistency in follow-up timepoints, with durations varying widely from 6 months to 8 years.
Indices	Various measurement methods were used to assess esthetic outcomes, making it difficult to draw definitive conclusions about the differences between IIP and DIP. For instance: Papillary recession ○ Direct periodontal probe reading ○ Calibrated photo-morphometry ○ Jemt papilla score ○ 3D surface superimposition Gingival recession PES
Molecular biology studies	A limited number of studies have attempted to evaluate biochemical changes related to implant healing. To date, existing research has identified dynamic changes in biomarker levels during osseointegration and differences associated with various implant loading protocols. However, there is a significant knowledge gap in this area; no study has investigated molecular activity specifically comparing between IIP and DIP yet.

## Data Availability

Not applicable.

## Supplementary Materials

The following supporting information can be downloaded at: https://www.mdpi.com/article/10.3390/jcm15020682/s1, Table S1: Preferred Reporting Items for Systematic reviews and Meta-Analyses extension for Scoping Reviews (PRISMA-ScR) Checklist [105].
